# Supplementary material for: Learning curve and surgical outcome of robotic assisted colorectal surgery with ERAS program
Source: Sci Rep. 2022 Nov 29;12:20566. doi: 10.1038/s41598-022-24665-w (PMC9709162; doi:10.1038/s41598-022-24665-w)
Supplement: Supplementary file 2 — Supplementary Information 2. [file 41598_2022_24665_MOESM2_ESM.zip › RAL Raw data-Ñ[▒K/114/OP_ñw╝╨░O▒Kñσ.pdf]

正式摘要(電子簽章：0000002261220054)

W83 / W83

臺中榮民總醫院

Operation Note

[redacted]

- 手術日期：2021/09/25
- 手術醫師：VS 蔣鋒帆，R 林志安，CR 陳映菁，R 謝亦婷
- Anesthesia : GE
- Aseptic agents : Betadine Alcohol , Betadine Aqua

Diagnosis

- Pre-operative diagnosis : Rectal tumor with liver metastasis, cT3N2M1a
- Post-operative diagnosis : Ditto.

Surgical Indication

- CURATIVE INTENDED AND STAGING,

Operation

- Robot Assisted Low Anterior Resection
  ILEOSTOMY

Finding

- ulcerative tumor, about 4x5 cm in size at 7 cm FAV.
  2. Total meso excision was complet
  3. IMA high ligation+
  4. distal margin 1 cm, Surefrom 45 X2 for rectum
  5. air tie test neg. donut intact.
  6. loop ileostomy in RLQ
  7. LATERAL PELVIC LAP AND DISSECTION WAS DONE BY GU DR YANG

Procedure

- 1. under GE, the pt was in lithotomy position
  2. the trocar hold was made in periumbilicus open method. the other 5 hole made by punctiure
  3. identify holly plain and dissct. isolated IMA root. ligated with clips
  4. dissect between Gerota fascia and meso rectum. lysis Lt white line
  5. dissect into pelvis area. dissect posterior side of rectum down to pelvis follor levetar ani. anterior side of Denovillor fascia. Total meso excision was complet
  6. resected rectum stump with Sure Form 45 2 fires.
  7. pull the specimen outside abdomen from midline wd
  8. embeded envril 29 mm for side to end
  9. resect S-colon by GIA 80/3.8mm
  10.reset daVinci scopy and complete anastomsis. air tie test neg. donut intact.
  11. loop ileostomy in RLQ
  12.he tolerate whole procedure and extubated.
- Estimated blood loss: minimum
- 器械數量: 正確
- 紗布數量: 正確

Image

- PAINT
  - colon & rectum

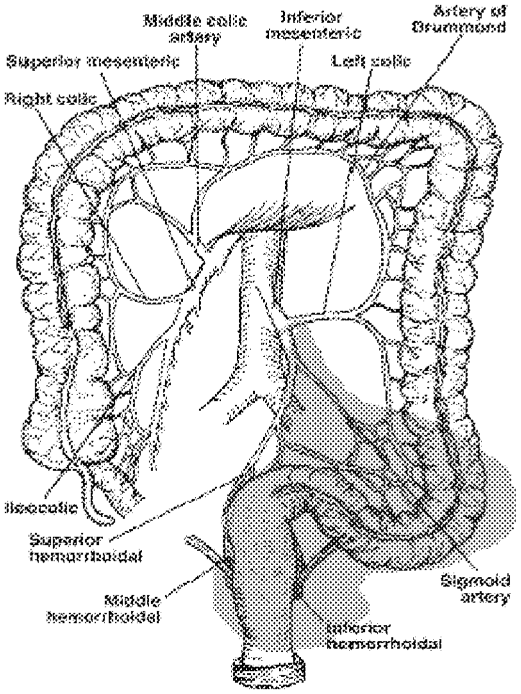

編輯主任 2062F蔣鋒帆

正式摘要(電子簽章：000000226122054)

W83 / W83

doctorRank:B
